# Feasibility Study of the World Health Organization Health Care Facility-Based Antimicrobial Stewardship Toolkit for Low- and Middle-Income Countries

**DOI:** 10.3390/antibiotics9090556

**Published:** 2020-08-29

**Authors:** Gina Maki, Ingrid Smith, Sarah Paulin, Linda Kaljee, Watipaso Kasambara, Jessie Mlotha, Pem Chuki, Priscilla Rupali, Dipendra R. Singh, Deepak C. Bajracharya, Lisa Barrow, Eliaser Johnson, Tyler Prentiss, Marcus Zervos

**Affiliations:** 1 Division of Infectious Disease, Henry Ford Health System, Detroit, MI 48202, USA; mzervos1@hfhs.org; 2 World Health Organization, 1202 Geneva, Switzerland; ismith@who.int (I.S.); paulins@who.int (S.P.); 3 Global Health Initiative, Henry Ford Health System, Detroit, MI 48202, USA; LKaljee1@hfhs.org (L.K.); TPRENTI1@hfhs.org (T.P.); 4 Ministry of Health, 207218 Lilongwe, Malawi; watipasokasa@yahoo.com (W.K.); rhodamgb@gmail.com (J.M.); 5 Jigme Dorji Wangchuck National Referral Hospital, 11001 Thimpu, Bhutan; pchuki@jdwnrh.gov.bt; 6 Department of Infectious Diseases, Christian Medical College, Vellore 632004, India; priscillarupali@yahoo.com; 7 Ministry of Health and Population, 44600 Kathmandu, Nepal; dipendra2028@gmail.com; 8 Group for Technical Assistance, 44600 Kathmandu, Nepal; bajra.deepak@gmail.com; 9 Department of Health & Social Affairs, 96941 Pohnpei, Federated States of Micronesia; lbarrow@fsmhealth.fm (L.B.); ejohnson@fsmhealth.fm (E.J.); 10 School of Medicine, Wayne State University, Detroit, MI 48202, USA

**Keywords:** antimicrobial resistance, antimicrobial stewardship, low- and middle-income countries, barriers and enablers

## Abstract

Antimicrobial stewardship (AMS) has emerged as a systematic approach to optimize antimicrobial use and reduce antimicrobial resistance. To support the implementation of AMS programs, the World Health Organization developed a draft toolkit for health care facility AMS programs in low- and middle-income countries. A feasibility study was conducted in Bhutan, the Federated States of Micronesia, Malawi, and Nepal to obtain local input on toolkit content and implementation of AMS programs. This descriptive qualitative study included semi-structured interviews with national- and facility-level stakeholders. Respondents identified AMS as a priority and perceived the draft toolkit as a much-needed document to further AMS program implementation. Facilitators for implementing AMS included strong national and facility leadership and clinical staff engagement. Barriers included lack of human and financial resources, inadequate regulations for prescription antibiotic sales, and insufficient AMS training. Action items for AMS implementation included improved laboratory surveillance, establishment of a stepwise approach for implementation, and mechanisms for reporting and feedback. Recommendations to improve the AMS toolkit’s content included additional guidance on defining the responsibilities of the committees and how to prioritize AMS programming based on local context. The AMS toolkit was perceived to be an important asset as countries and health care facilities move forward to implement AMS programs.

## 1. Introduction

The misuse of antimicrobials is one of the main drivers for the development of antimicrobial resistance (AMR) [1,2]. Antimicrobial stewardship (AMS) programs have been shown to be effective in reducing unneeded antimicrobial use and slowing AMR in high-income countries; however, there are limited data on the feasibility of AMS programs in low- and middle-income countries (LMIC) [3,4,5,6]. As a response, the World Health Organization (WHO) has developed a practical toolkit for health care facility-based AMS programs in LMIC (hereafter referred to as the “AMS toolkit”) [7].

In 2015, at the 68th World Health Assembly, AMR was recognized as a threat to public health. A global action plan, including an objective to optimize the use of antimicrobials, was endorsed during the Assembly. AMS programs aim not only to optimize antimicrobial use, but also to improve patient outcomes, decrease rates of AMR, and reduce health care costs [8,9,10,11]. With few new antimicrobials being produced and the decreased effectiveness of existing antimicrobials, AMS programs are an essential component of a One Health approach to address AMR [12]. Many LMIC have inadequate AMS policies and treatment guidelines at both the national and health care facility levels, resulting in a disproportionate impact of AMR in these countries [1,2,11,13,14,15]. Intraregional, interdisciplinary collaborations and partnerships are needed at the national and facility level to adapt, implement, and disseminate AMS programs that are locally salient [16,17,18].

The AMS toolkit is divided into six sections: (1) Structural Core Elements for AMS Implementation at the National Level; (2) Structural Core Elements for AMS Implementation at the Facility Level; (3) Planning AMS programs; (4) Performing AMS interventions; (5) Assessing AMS programs; (6) Education and Training. As part of the AMS toolkit’s development, a feasibility study was undertaken in Bhutan, the Federated States of Micronesia (FSM), Malawi, and Nepal. The study objectives were to (1) assess local knowledge and perceptions regarding AMR, AMS, and key concepts of the AMS toolkit; (2) identify barriers and facilitators for the implementation of AMS programs and policies; (3) identify recommendations to revise the AMS toolkit draft to ensure it meets the needs of a broad range of LMIC settings; (4) to provide recommendations to the countries on initiating and strengthening AMS programs. The study countries were selected based on geographic regions where AMR is a significant issue and where, to date, there are limited resources and AMS programs. These countries also represent different regions and varying healthcare systems (Table 1).

## 2. Results

The following results are organized by national and health care facility core elements and health care facility-based interventions as described within the AMS toolkit. In addition, we have included data on actionable items and recommendations for the draft toolkit. A summary of demographic information is found in Table 2.

Key findings from this study are shown in Table 3.

### 2.1. National Core Elements

The national core elements include (1) National Plan and Strategies; (2) Regulations and Guidelines; (3) Education and Training; (4) Supporting Technologies and Data.

#### 2.1.1. National Plan and Strategies

All study countries identified AMR as a growing threat and have drafted an AMR National Action Plan (NAP); however, the countries were at various levels in terms of NAP implementation. Relatively few respondents outside of the national government were aware of the NAP content.

**Remark** **1.** *“I have heard of the National Plan, but it is not being implemented in our medical college….” (Hospital Administrator, Nepal)*

Study participants aware of the NAP reported dedicated high-level national leadership in support of the NAP. In addition, some countries have obtained outside funding to support portions of the NAP. However, across the four countries, numerous barriers were identified that affected the implementation of NAP policies and programs. These barriers included the need for additional financial support and technical assistance, limited laboratory capacity, including infrastructure and expertise, and lack of technical expertise on AMR-specific issues.

**Remark** **2.** *“…again, it involves a lot of resources. You need to see the sensitivity and (do) surveillance …to review sensitivities…A lot of budget is involved in that. So, funding is another part (in terms of) feasibility or not. And of course, the human side and human resources are also important....” (National Level, Nepal)*

#### 2.1.2. Regulations and Guidelines

Facilitators for AMR regulations and guidelines included the existence of drafted standard antibiotic prescribing guidelines, which have been developed in Bhutan, Malawi, and Nepal. In FSM, there is an antibiotic prescribing guideline that has been reviewed with national stakeholders and external technical expertise. FSM, Malawi, and Nepal are in the process of developing and implementing policies for prescription-only antibiotic sales. In Bhutan, prescription-only regulations are implemented and enforced. The key barriers identified included inadequate monitoring and evaluation at the national and regional levels for infection prevention and control (IPC) and AMS including adherence to guidelines, inadequate laboratory facilities to provide empiric diagnostic data and support, implementation of the AWaRe (Access, Watch, Reserve) classification of antibiotics, challenges with the supply chain of medications in hospital pharmacies, and lack of prescribers (physicians) in remote areas.

**Remark** **3.** *“….I’m not claiming that just writing in law will be sufficient to really restrict prescriptions for selling…we have so many pharmacies which have been already selling without prescriptions… we need to really go and then take action against those who are selling the antibiotics…at the same time in the public sector we are promoting some antibiotics to be used by [non-physicians]…because neonatal mortality [is] very high…” (National Level, Nepal)*

**Remark** **4.** *“…If we don’t have the drugs [in the hospital], the doctor will prescribe and then the patient is obligated to buy in a pharmacy….” (National Level, Malawi)*

#### 2.1.3. Awareness, Training, and Education 

At the national level, there was strong support to enhance education and training in AMR/AMS for physicians, nurses, pharmacists, and laboratory staff. At the broader community level, AMS awareness, training, and education included public antibiotic information campaigns. Barriers included lack of dedicated financial support and limited training and technical expertise. Respondents also reported the need for more community advocacy and awareness of AMR.

**Remark** **5.** *“…and we are also thinking to develop some sort of dramatizing…learning materials and publishing to the media (for the community). So that type of materials we’re planning to do. That requires a little bit budget so we are constrained with budget so we’re planning anyway…and also planning to do some training for health professionals…primary, community levels–upper-level health professionals also need to have training….” (National Level, Nepal)*

#### 2.1.4. Supporting Technologies and Data

Bhutan, Malawi, and Nepal are recipients of Fleming Fund grants to expand microbiology laboratory capacity and strengthen surveillance systems [16]. Respondents perceived this support as a starting point to significantly enhance NAP implementation. However, respondents also recognized the need for more sustained funding and training in diagnostic testing, laboratory surveillance, and information technologies, and the struggle with inadequate local expertise in monitoring antimicrobial use and consumption.

**Remark** **6.** *“So, we are so glad that at least one of our key priorities for the NAP–AMR surveillance—we have a country grant to support improving lab capacity and surveillance. So, that’s a very good plus for us….” (National Level, Malawi)*

### 2.2. Health Care Facility Core Elements 

Health care facility core elements include (1) Leadership Commitment; (2) Accountability and Responsibilities; (3) Education and Training; (4) Monitoring and Surveillance; (5) Reporting and Feedback. 

#### 2.2.1. Leadership Commitment 

A key component of facility-based AMS is dedicated support from facility leadership to encourage the development of AMS and IPC committees and implement programs. Facility leaders stated that the recommended AMS activities in the toolkit are feasible and necessary to decrease AMR. Barriers to leadership commitment included inadequate dedicated human and financial resources for AMS programs, and inadequate internal communication between administrators, management, and staff.

**Remark** **7.** *“A successful stewardship program can only work when there is good collaboration between top management and middle-level management. Because middle-level management, they are the ones that are in touch with the staff on the ground there.” (Hospital Administrator, Malawi)*

**Remark** **8.** *“Stressing the importance of leadership commitment is really important. Because nothing really happens without leadership commitment.” (National Level, FSM)*

#### 2.2.2. Accountability and Responsibilities

Respondents described active IPC committees, quality improvement teams, and drug and therapeutic committees. These committees are engaged in routine activities and provide important feedback and training to clinical personnel. Building on these committees, leaders and clinical staff were enthusiastic about future implementation of AMS committees and antibiotic prescribing guidelines.

**Remark** **9.** *“…we don’t have an AMS team but the infection control committee they have nurse staff. They do regular monitoring of cultures of different areas of the theatre…they take a routine culture on a regular basis….” (Hospital Administrator, Nepal)*

Barriers included physicians’ reluctance to change their prescribing practices, even with the available evidence-based guidelines, and provider-heavy workloads, which decreased interest in devoting time to an AMS committee.

**Remark** **10.** *“We have an issue of shortages of staff…we also have issues of people wearing too many hats, so that is part of, I guess, it’s kind of like we’re not sure who’s going to take care of it, and wonder who which program is.” (Clinical Staff, FSM)*

#### 2.2.3. Education and Training

There was a high level of enthusiasm for expanding AMS education and training within health care facilities. Currently, some study facilities have an established education infrastructure (e.g., continuing medical education) that can support additional AMR/AMS training. However, despite enthusiasm, time and resources were barriers. Many facilities lack space and available expertise to support trainings. At some sites, even IPC trainings are limited due to inadequate resources. In addition, some respondents emphasized the need for a ‘hands-on’ approach to support sustained knowledge.

**Remark** **11.** *“Major barriers as of right now is lack of knowledge mainly. We have high staff turnover, so in the last three years there have been no new trainings. Almost 75% of the staff may not be aware of the IP (infection prevention) practices….” (Clinical Staff, Malawi)*

**Remark** **12.** *“So hands-on is a very, very important part of it. As compared to just reading and looking at the modules. But you have to apply, the application of that knowledge needs to be implemented as well. With the hands-on skills I think it will stick and will stay there longer, and be more useful to the people. In my opinion, I think two or more models of education is probably the best suitable for us in this setting.”*

#### 2.2.4. Monitoring and Surveillance

Among microbiologists and laboratory staff, there was a strong desire for capacity building and international support for up-to-date equipment and supplies and development of AMR surveillance systems. However, in most health care facility sites, there was inadequate capacity to conduct point prevalence surveys and routine surveillance of susceptibility patterns, including health care facility-specific antibiogram data.

**Remark** **13.** *“For the lab, our major challenges are both human and material resources…we have a very big challenge procuring laboratory microbiology supplies. Either maybe because of the budget or our major supplier—the central medical stores—they don’t have them in stock……we have the knowledge, but resources are not there….” (Clinical Staff, Malawi)*

#### 2.2.5. Reporting and Feedback

In health care facilities with developed IPC committees, there are existing reporting and feedback structures in place that can be expanded to include AMS. However, in most facilities, there are inadequate structures for reporting these data to facility management and clinical staff. Study participants noted there was little communication between laboratory and clinical personnel, decreasing opportunities for information exchange about AMR patterns and impact of the use of specific antibiotics.

**Remark** **14.** *“…We are very much lacking in reporting and feedback. We can do something but this is one area we really have to really have to think and discuss with regards to core elements…there are so many challenges, which we have to sit together and discuss and see how to move things forward.” (Clinical Staff, Bhutan)*

### 2.3. Action Items at the National and Health Care Facility Level

Multiple action items were identified at both the national and facility level to move forward with implementation of the NAP and the WHO toolkit and development and implementation of AMS committees and other supporting programs and policies. Identification of sustainable funding and technical expertise in human, animal, and environmental health was essential across each of the core elements. Action items at the national and facility level are found in Box 1 and Box 2.

Box 1 **National-level action items to support AMS and toolkit implementation.**

- Establish terms of reference for National AMR technical working groups.
- Perform needs assessments of local laboratory capacity at the national and local levels.
- Update National Essential Medicine Lists or equivalent documents, including integration of the WHO AWaRe categories.
- Sensitize health care providers about AwaRE categories.
- Review, update, and implement national and district/state/regional antibiotic prescribing guidelines informed by available AMR surveillance data.
- Develop needed resources for health care facility leadership to ensure antibiotic prescribing guidelines are followed consistently across the country.
- Increase national antibiotic awareness campaigns.
- Develop or expand age-relevant education AMR/AMS programs in public school systems.
- Strengthen microbiology laboratory capacity and expand training to facility-based laboratory staff to support and encourage engagement in AMS and national surveillance.

AMR—antimicrobial resistance; AMS—antimicrobial stewardship.

Box 2 **Health care facility-level action items to support AMS and toolkit implementation.**

- Identify funding sources to support facility-level AMS.
- Sensitize facility leaders about the urgency of AMR as a health risk.
- Increase facility leaders’ awareness of National Action Plan (NAP) content, government roll out plans, and potential funding and resources to support facility-based AMS.
- Develop stepwise approaches to implement AMS considering facility capacities throughout the country.
- Standardize IPC committee roles and responsibilities.
- Identify dedicated leaders and champions within facilities who will take responsibility for establishing AMS committees and implement AMS programs. In many instances, individuals involved in IPC, QIT, and DTC committees can serve as key stakeholders in this process.
- Develop/adapt standard antibiotic prescribing guidelines informed by local AMR surveillance data patterns.
- Strengthen laboratory capacity to ensure annual output of aggregate antibiograms and support regular reporting to national laboratories for AMR surveillance.
- Establish mechanisms for reporting and feedback on the implementation of AMS interventions and adherence to antibiotic prescribing guidelines based on international consensus and local input.
- Integrate AMS training into existing CME programs and IPC training initiatives across all health disciplines.
- Develop interdisciplinary training programs to support increased understanding and communication between wards and departments.
- Develop training-of-trainer workshops on AMS and cascade training to other health care providers in the health care facilities.

AMR—antimicrobial resistance; AMS—antimicrobial stewardship; CME—continuing medical education; DTC—drug and therapeutic committees; IPC—infection prevention and control; QIT—quality improvement teams.

### 2.4. Health Care Facility-Based AMS Interventions

The WHO toolkit provides a detailed overview of evidence-based health care facility AMS interventions including (1) persuasive, educational, and feedback; (2) restrictive; (3) structural interventions. The toolkit also includes information on planning, implementing, and assessing AMS programs. Identified enablers to support AMS interventions included strong leadership support at the health care facility administration level, overall strong interest in education and training in IPC, AMR, and AMS among clinical staff, and perceptions among staff that AMS interventions decrease unnecessary antibiotic use. Barriers included inadequate local infectious disease, AMR and AMS expertise, and limited financial and human resources to implement interventions and conduct program monitoring and evaluation.

**Remark** **15.** *“When it comes to interventions I think they are very much appropriate because many of these problems do exist in our…day-to-day practices. But we are not really assessing them…to the fullest extent about the interventions.” (Clinical Staff, Bhutan)*

### 2.5. Summary of Recommendations for the Draft WHO Toolkit

Study participants suggested recommendations on the improvement of content, organization, and presentation of materials, which were incorporated into the final version of the WHO AMS toolkit (Table 4). Participants also noted that the review of the AMS toolkit needs to be an iterative process as implementation of the toolkit and AMS programs and policies progress in each country.

**Remark** **16.** *“I think it’s a good start. It has more stuff, areas that need to be stringent, if we kind of have an impact on this issue. And I think it’s pretty comprehensive in a sense… But I think it should be an organic process, as we move along and identify issues, we address them and continue to make improvements.” (Clinical Staff, FSM)*

## 3. Discussion

The toolkit was universally well-received by policy makers, facility management, and clinical staff levels throughout the four study countries. Data were obtained from a diverse multinational and interdisciplinary group of stakeholders. Identification of possible enablers and barriers for toolkit implementation at the national and facility level supported revisions to ensure that the toolkit meets the needs of a broad range of LMIC settings. Each study country presented different contextual factors to consider regarding AMS implementation and use of the AMS toolkit. Varying factors included different health priorities at both the national and facility level, current status of nationalized universal health care plans, variances in public health funding, availability and use of antibiotics, and development and enforcement of prescription-only regulations. These factors must be considered on a country-by-country basis for stakeholder engagement and evaluating pathways to toolkit implementation.

Key facilitators and enablers included strong leadership commitment at the national, local, and facility levels, increases in funding mechanisms to support development of surveillance systems within countries, and increased awareness of AMR. Key barriers to AMS implementation included limited human and financial resources, inadequate supporting technologies (e.g., monitoring and surveillance), and communication challenges between facility administration and staff, and between staff members. These barriers can be mitigated using a clear step-by-step approach, as indicated in the WHO AMS toolkit, tailored to specific country and facility contexts and needs. In addition, a multidisciplinary training and education approach can potentially strengthen AMS commitment and communication within health care facilities. 

Prior studies have described the core elements of AMS programs in LMIC settings, including the need to build laboratory capacity, enhance IPC, and establish surveillance systems of both infections and antibiotic use [15,19,20,21,22,23,24,25]. Feasibility study data support these needs, as well as other essential elements of AMS. Overall, respondents stated that leadership support at the national and senior facility management levels was needed for successful implementation. In addition, stepwise implementation strategies were universally considered to be useful. At the national level, respondents supported the urgent need for the development and implementation of antibiotic treatment guidelines. AMS must be identified as a national priority and included in facility key performance indicators, requiring dedicated support, accountability, and assigned roles and responsibilities. Within health care facilities, AMS must include written strategies, implementation of a formal multidisciplinary structure including laboratory surveillance and IPC, and identification of dedicated staff with clearly defined roles. These strategies must include support and expertise on infection management, access to timely laboratory/imaging/information technology services and available trained and experienced professionals in AMR and infectious disease.

Respondents reported the need for increased and consistent education and training. A first step towards strengthening educational initiatives includes understanding current clinical staff competencies and building tailored projects and programs that emphasize and develop knowledge and skills. Education must be ongoing, hands on, and practical, and include incentives and a broad range of resources (e.g., face-to-face and web-based).

Respondents felt the toolkit provided important information on appropriate antibiotic use and consumption and the means to utilize less expensive and technologically based approaches appropriate to LMIC contexts. AMS committees were considered at the heart of effective facility-based stewardship. Therefore, these committees must be formed and members must receive regular training in antimicrobial prescribing practices and stewardship. The committee should be responsible for reviewing and auditing courses of therapy for specified antimicrobial agents and clinical conditions. There is also the need for an established and effective communication strategy between the AMS committee, leadership, and clinical staff.

In terms of health care facility-based AMS, evidence-based antibiotic treatment guidelines were identified as a key component of AMS. Where possible, guidelines should be based on local antibiotic resistance patterns and availability and cost-effectiveness of agents. Day-to-day guidelines should be kept simple and include empiric antibiotic selection, definitive antibiotic selection, organism and disease states, intravenous to oral conversion, renal dosing, and duration of therapy. In conjunction with those guidelines, AMS programs are needed to reduce the overuse and overprescribing of antibiotics, the use of broad-spectrum antibiotics and dose combinations, and delayed prescribing.

Health care facilities in LMIC need capacity building both in terms of human and technological resources to develop a formulary and auditing process, antibiotic prescribing documentation policies and procedures, and regulations regarding drug restrictions including use of the WHO AWaRe (Access, Watch, Reserve) classifications. Health care facility monitoring and surveillance capabilities need to be developed to support AMS initiatives including measures to monitor quality/quantity of antimicrobial use at the unit and facility-wide level, compliance with specific interventions, and identification of antibiotic susceptibility rates for locally significant pathogens. 

### Limitations

The feasibility study was conducted in only four countries with a sample size of 12 national leaders, 21 facility administrators, and 65 clinical staff. Only one country was selected from Africa and there were no LMIC from Latin America, the Caribbean, the Middle East, or Europe included. Despite the small sample size, purposeful sampling was undertaken to ensure that different regions of the study countries were included with a diverse group of national- and facility-based stakeholders. These data provide a general overview of barriers and facilitators for implementation of AMS programs and the AMS toolkit. As implementation of the toolkit moves forward, additional data from other countries will continue to contribute to future versions. In addition, the feasibility study was focused on health facilities which provide inpatient care. Respondents discussed the need for AMS within community health facilities and education for patients. Future research and development of community-based AMS training and interventions are needed to address the high consumption of antibiotics outside of inpatient facilities.

## 4. Materials and Methods

### 4.1. Overview

The feasibility study was conducted from February 2019 to May 2019. The project was a partnership inclusive of a multinational and interdisciplinary team with expertise in AMR and AMS, infectious diseases, IPC, public health, nursing sciences, pharmacy, and social sciences. The study countries were selected by both WHO staff and the HFHS feasibility study team, based on geographic regions where AMR is a significant issue and where, to date, there are limited resources and AMS programs. These countries represent diverse contexts and challenges associated with the implementation of health care facility-based AMS programs. In addition, WHO and/or HFHS had worked with AMS leaders in the four selected countries, which facilitated rapid implementation of the feasibility study.

The study population included national- and local-level policymakers, facility administrators, and clinical staff. Study health care facilities were identified by in-country investigators and coordinators, and represented various facilities (e.g., public, private, and non-profit) and diverse geographic regions within each country. All facilities included inpatient care and ranged in size from 36 to 850 beds. Participating clinical staff included physicians, nurses, pharmacists, microbiologists, and laboratory technicians.

The study used a qualitative design based on key domains for program feasibility studies [26,27]. These included (1) acceptability of the toolkit; (2) demand and anticipated use; (3) practicality of the toolkit for use in LMIC; (4) integration of the toolkit within existing infrastructures; (5) adaptability of the toolkit within local contexts; (6) implementation and dissemination enablers and barriers to toolkit sustainability and scale-up within LMIC. The qualitative approach provided opportunity to engage with multiple partners from the selected sites throughout the development, implementation, and dissemination of the study. Through this engagement, conversations about AMR during meetings, workshops, and interviews provided visibility to local, national, and international issues related to AMR, the role of stewardship in the contexts of LMIC, and the potential for adaptation of the WHO toolkit to support AMR stewardship at the policy and programmatic levels in multiple settings.

In each country, the project was undertaken after an initial meeting with in-country study investigators and local governmental, nongovernmental, and health care facility stakeholders. After completion of the study, dissemination stakeholder workshops were convened within each country. These workshops provided opportunity for local input on (1) the interpretation of the feasibility study data; (2) ways to address facilitators and barriers to implementation of the AMS toolkit and AMS programs and policies; (3) identification of actionable items to promote implementation. This input from each country is reflected in the final reports and subsequently, in this paper.

### 4.2. Sample Size and Recruitment

Overall, 12 policy makers were recruited and interviewed, and 15 health care facilities were selected between the four countries. With the facilities, a total of 21 administrators, 20 physicians, 21 nurses, 11 pharmacists, and 13 laboratory personnel were interviewed (Table 5). When the study started, the research team estimated the sample size with the stipulation that it could be smaller or larger depending on data saturation. In each site, the team felt confident that the data collected reached saturation and no significant additional information was being recorded to justify additional interviews.

National-level respondents were identified by in-country principal investigators and coordinators as well as recommendations from the WHO Country Offices and included individuals involved in the development of AMS NAP and other experts in AMR and AMS. At the facility level, administrators or managers were invited to participate in the study. Clinical staff selection criteria included individuals engaged in current or past IPC programs and/or those engaged in existing AMS committees. A range of staff were interviewed, including ward physicians and nurses, laboratory staff, and pharmacists. In each country, the international and local partners worked closely together to approach potential respondents to explain the purpose of the study and request their participation. At the policy and hospital administration level, all of those approached made themselves available for the interviews. At the clinical level, the study team requested interviews with representatives from nursing, medicine, pharmacy, and microbiology/laboratory staff (Table 5). Potential participants were provided with an abbreviated version of the toolkit with sections specific to their position (e.g., policy maker, administrator, and staff) and a list of key topics to be covered in the interview [28].

### 4.3. Research Instruments

Three interview guides and demographic forms were developed specific to the population groups (policymakers, health care facility administrators, and clinical staff). Draft interview guides and demographic forms were provided to in-country investigators to review and revise to ensure they reflected local contexts. Interview guide items and probes focused on the research objectives, the toolkit core elements for health care facility-based AMS in LMIC, and the 6 program feasibility key domains. Demographic forms included items on current institutional affiliation (e.g., city/district, type of institution, and number of beds), respondents’ education and employment (e.g., current position and years in current position), and engagement in AMR, AMS, and IPC programs at the health care facility or national levels (see Table S1).

### 4.4. Data Collection and Management

Data collection was led by team members from the Henry Ford Health System in partnership with local staff. In Bhutan, additional data collection support was provided by investigators/infectious disease specialists from Christian Medical College, Vellore, India. Interviews were conducted in English, with interpretation to local language verbally as required. Interviews were audiotaped and transcribed. Transcribed data were entered into Ethnograph, version 6, a qualitative data management software. A data coding dictionary was developed based on the interview guides and emergent themes from team members’ field experiences and the transcribed text.

### 4.5. Data Analysis

After initial coding was completed, groups of code words were organized under common topics including the AMS core elements, the key domains for the feasibility study, and emergent themes. Searches were conducted within common themes, study country, and population (policymakers, administrators, and staff). Search documents were saved and reviewed to identify key findings and recommendations within and across countries in terms of barriers and facilitators for health care facility-based AMS policies and programs, action items to strengthen AMS policies and programs including implementation of the AMS toolkit, and specific recommendations for the AMS toolkit’s content and organization. Illustrative text within the transcripts were identified to support the summary conclusions. Country-specific draft reports were sent to in-country investigators and other stakeholders for their review and input.

### 4.6. Ethical Approval

The project was approved by ethical review boards in each country, the Henry Ford Health System Institutional Review Board (Detroit, MI, USA), and the WHO Ethical Review Committee (Geneva) (Approval February 2019, #ppp3131/004479)]. All participants provided written informed consent.

## 5. Conclusions

There was clear consensus that optimal implementation and use of the toolkit requires recognition of country-specific contexts. These include diagnostic challenges, laboratory capacity, and high burdens of infectious diseases. Health care workers responsible for prescribing antibiotics have a broad range of education, training, and experience. Development of antibiotic prescribing guidelines may often be limited by inadequate local data on disease burden and susceptibility patterns. Many LMIC have poorly regulated prescription-only policies or limited access to essential antibiotics

Despite these challenges, the consensus among study respondents was that the toolkit will be an important asset as countries and health care facilities move forward to combat AMR and implement AMS programs. More information will be needed to address implementation strategies and many barriers need to be addressed to increase likelihood of successful implementation within the study countries and other LMIC. The road ahead must include commitment at the national and facility levels to prioritize AMR and develop sustainable national and local initiatives. The WHO toolkit provides a comprehensive review of core elements of AMS, strategies for intervention adaptation and implementation, and expansion of training and educational platforms. With growing global concerns regarding AMR, the WHO toolkit can provide practical guidance and support to LMIC worldwide [1].

## Figures and Tables

**Table 1:** Information on Health Care Systems, AMR Stewardship, and Pharmaceutical Sales by Country.

Country/Population	Country-Specific Details
Bhutan 727,000	**Health Care System:** Majority public funded health system with some private providers. The National Health System provides free health care, including pharmaceuticals. **AMR Stewardship:** The Bhutan NAP on AMR was launched by the Bhutan cabinet in May 2017. AMS is prioritized in the NAP; a hospital-based AMS program has been initiated and the National Referral Hospital will function as the National AMS Coordination Center. **Pharmaceutical Sales:** Sales of antimicrobials by prescription-only is regulated by the Drug Regulatory Authority and the ban of irrational fixed-dose combinations (FDCs) is consistent with the WHO restricted list of FDCs.
Federated States of Micronesia (FSM) 100,000	**Health Care System:** There is one public hospital within each of the four island states and a fifth private hospital on the island of Pohnpei. The Department of Health Services in each state provides medical and public health services through a hospital, community health centers, and dispensaries. Each state system is autonomous. Health services are highly subsidized by the state governments, except in private clinics. There are six private health clinics in the country and one private hospital. Transportation difficulties between islands often prevent outer island residents from accessing hospital services.^1^ **AMR Stewardship:** The NAP on AMR in FSM has been drafted, but not yet implemented country-wide. A technical working group (TWG) comprised of national-level policymakers has worked on the implementation of the NAP, but it has not yet been endorsed by Congress. An AMS program across hospitals and other community health facilities has been identified as a priority in the NAP. Representatives from each of the four states have also worked on the revising and editing of the NAP to make it applicable across the entirety of FSM. **Pharmaceutical Sales:** A bill on prescription-only regulation of all antibiotic sales is currently under advisement in the National Congress. Many patients within FSM are served by community dispensaries, particularly in the outer islands of the region where few qualified healthcare professionals are practicing. These dispensaries are undergoing a review of standards. Antibiotic purchasing is handled at the state level, guided by the National Essential Medicines List. When antibiotic inefficacy is suspected, quality control testing is undertaken in connection with the Therapeutic Goods Administration in Australia.
Malawi 18.6 million	**Health Care Services:** Health services in Malawi are provided by public, private for profit (PFP), and private not for profit (PNFP) sectors. Health services in the public sector are free-of-charge at the point of use. The PFP sector consists of private hospitals, clinics, laboratories, and pharmacies. Traditional healers are also prominent and would be classified as PFP. The PNFP sector comprises of religious institutions, nongovernmental organizations (NGOs), statutory corporations and companies.^2^ **AMR Stewardship:** In 2015, a situational analysis of AMR was undertaken by the Ministry of Health. In 2017–2018, a NAP on AMR based on a One Health approach was developed and approved. **Pharmaceutical Sales:** Regulations to restrict nonprescription sale of antibiotics are limited and antibiotics are readily available in communities throughout Malawi. There are national-level guidelines, which were implemented in 2014; however, there is a need for revision to reflect the specific patterns of resistance throughout Malawi. A majority of antibiotics are prescribed without any definitive laboratory data on pathogen or resistance. Hospitals are dependent on donations for many pharmaceuticals, including antibiotics, and certain antibiotics may be overprescribed because of limited options.
Nepal 26 million	**Health Care System:** Nepal’s health system includes public, private, and not-for-profit facilities. As part of the new federalist government system’s restructuring, the public health system is being decentralized, with 16 tertiary hospitals being managed by the federal government and primary and secondary hospitals being managed at the provincial level. At the same time, the Ministry of Health and Population (MOHP) is expanding access to Universal Health Care throughout the country. **AMR Stewardship:** The MOHP has also established a multisector AMR Steering Committee inclusive of a TWG, which has been approved by the Deputy Prime Minister. As of 2019, a NAP on AMR has been drafted and includes AMS as a priority. **Pharmaceutical Sales:** Regulations to restrict nonprescription sale of antibiotics are limited, with little monitoring and enforcement of existing policies. Many remote areas do not have access to trained physicians, so other health providers must dispense antibiotics in public health centers.

^1^ World Health Organization Western Pacific Region. 2017. Federated States of Micronesia: WHO Country Cooperation Strategy, 2018-2022. ^2^ Ministry of Health and Population, Republic of Malawi. The Health Care System. Available at: https://www.health.gov.mw/index.php/2016-01-06-19-58-23/national-aids.

**Table 2:** Clinical Staff Demographics.

Demographic Description		Bhutan	Federated States of Micronesia	Malawi	Nepal
**Total staff interviewed**	-	16	21	16	12
**Members of IPC committee**	Yes	10	5	9	6
	No	6	12	2	5
	No IPC at institution	-	3	3	1
	No response	-	1	2	-
**Average years at institution**	-	8.0 (range 2–19)	14.2 (range 0.33–30)	7.2 years (range 0.75–14)	15.4 (range 1–35)
**Average years working on AMR**	-	6.9 (range 1–28)	9.8 (range 1–26)	5.1 years (range 0.33–21)	6.4 (range 1.5–20)
**Facility classification**	Public	16	20	16	5
	Private	0	1	0	4
	Non-profit	-	-	-	3

AMR—antimicrobial resistance; IPC—infection prevention and control.

**Table 3:** Key findings for Implementation of AMS in LMIC.

Implementation Category	Key Findings
**AMS implementation facilitators**	Strong national and health care facility leadership. Clinical staff engagement in AMS committees.
**AMS implementation barriers**	Inadequate human and financial resources. Limited supplies of antibiotics, particularly in remote regions. Lack of enforcement of regulations for prescription-only sales of antibiotics. AMS competencies among health care workers and limited training and education in AMR, AMS, and IPC.
**Recommendations to strengthen health care facility-based AMS**	Dedicated financial resources and AMS leaders and champions. Use of stepwise approaches for AMS implementation based on country and health care facility contexts. Mechanisms for reporting and feedback. Implementation of interdisciplinary AMS training workshops and AMS curricula.

AMR—antimicrobial resistance; AMS—antimicrobial stewardship; IPC—infection prevention and control.

**Table 4:** Key recommendations and implemented changes in the WHO AMS toolkit

Study Participants’ Recommendations	Specific Changes to Toolkit	Toolkit Reference
Easy-to-follow directions in terms of which chapters were most relevant for specific audiences	Key target audience was added	Top of first page of all chapters
Additional information on how to prioritize AMS activities (short-, medium-, and long-term) and guidance on stratification of interventions and assessment procedures based on local resources. Guidance in prioritizing AMS activities based on available resources, establishing stronger linkages between existing programs, e.g., IPC and AMS, and instituting the roles and responsibilities of members of AMS committees.	Key steps in establishing a national AMS program to enable facility AMS;	Ch. 1, Page 3, Box 1
	Key steps to establishing a health care facility AMS program;	Ch. 1 Page 4, Box 2
	Indicators from the Tripartite M&E framework for the Global Action Plan on AMR relevant to AMS programs;	Ch. 2, Page 10, Table 3
	Preparation for developing and implementing an AMS program in a health care facility;	Ch. 4, Page 18, Table 5
	Sample AMS review form.	Page 67, Annex IV
Definition of the role and function of an AMS champion. Definition of roles within AMS interventions for various types of health providers (e.g., physician, nurse, and microbiologist).	Sample terms of reference national AMS technical working group;	Page 63, Annex I
	Sample terms of reference health care facility AMS committee;	Page 64, Annex II
	Sample terms of reference health care facility AMS team.	Page 66, Annex III
Information or resource links that can guide countries in the development of AMS and AMR antibiotic prescribing guidelines in regions without hospitals and physicians.	Snapshot of GLASS;	Ch. 4, Page 29, Box 7
	Sample pre-authorization/restricted prescribing form;	Page 68, Annex V
	Sample medical chart;	Page 69, Annex VI
	Sample bug–drug chart;	Page 70, Annex VII
	Sample cumulative antibiogram for Gram-negative bacteria;	Page 71, Annex VIII
Training information to support effective AMS and IPC committees in terms of leadership skills, division of staff roles and responsibilities, reporting and feedback systems, and interdisciplinary communication.	Core components of IPC and the link to AMS;	Ch. 4, Page 23, Box 4
	Step-by-step guide for setting up an AMC surveillance program at the facility level;	Ch. 4, Page 25, Box 5
	Step-by-step guide for setting up a health care facility PPS;	Ch. 4, Page 26, Box 6
	The quality improvement model in more detail;	Ch. 5, Page 34, Figure 15
	Core steps for implementing an educational program.	Ch. 7, Page 60, Box 9

**Table 5:** Study sites, health care facility types, and sample sizes for policy makers, facility administrators, and clinical staff.

Country	Location	Facility	Policy Makers	Administrators	Staff
**Bhutan**	Central	Public	3	5	6 physicians 4 nurses 3 pharmacists 3 laboratory
	Western	Public			
	Eastern	Public			
**FSM**	Chuuk State	Public	3	7	8 physicians 6 nurses 2 pharmacists 4 laboratory
	Kosrae State	Public			
	Pohnpei State	Public			
	Yap State	Public			
**Malawi**	Lilongwe	Public	3	5	3 physicians 6 nurses 4 pharmacists 4 laboratory
	Lilongwe	Public			
	Mzuzu	Public			
	Blantyre	Public			
**Nepal**	Kathmandu	Non-profit	3	4	3 physicians 5 nurses 2 pharmacists 2 laboratory
	Kathmandu	Public			
	Nepalgunj	Private			
	Dharan	Private			
**TOTAL**	-	-	12	21	20 physicians 21 nurses 11 pharmacists

## Supplementary Materials

The following are available online at https://www.mdpi.com/2079-6382/9/9/556/s1, Table S1. Interview guides.
