# Supplementary material for: Feasibility Study of the World Health Organization Health Care Facility-Based Antimicrobial Stewardship Toolkit for Low- and Middle-Income Countries
Source: Antibiotics (Basel). 2020 Aug 29;9(9):556. doi: 10.3390/antibiotics9090556 (PMC7558985; doi:10.3390/antibiotics9090556)
Supplement: Supplementary file 1 [file antibiotics-09-00556-s001.pdf]

Supplementary Material

Table S1. Interview guides.

Policy Makers	Hospital Administrators	Hospital Staff
**Overall Introduction:** What role does your institution play in supporting hospitals in the implementation of antimicrobial stewardship and IPC programs? What is your role?	**Overall Introduction:** What are your roles and responsibilities at this hospital in terms of AMR stewardship? **Probes:** liaison with national leaders in AMR/stewardship, education and training, providing technical support, developing and supporting use of guidelines, supervising stewardship interventions,supporting/providing resources (human, financial), etc	**Overall Introduction:**What are the roles and responsibilities of [physicians, nurses, pharmacists, microbiologists/laboratory staff – **choose based on respondent position]** in terms of AMR stewardship?**.** What are your roles and responsibilities in terms of AMR stewardship? **Probes:** education and training, providing technical support, AMR surveillance (antibiograms, diagnostics),supporting use of guidelines, supervising stewardship interventions,participating in stewardship efforts, part of the infection control committee (ICC) or drug and therapeutics committee (DTC) etc
**Introduction/review of the toolkit and the core elements**<br>Chapter 2 on Structures; the introduction and the national core elements<br>Chapter 6 on Education and Training: The introduction and abbreviated tables for AMS competencies and workshop	**Introduction/Review of the toolkit and the core elements**<br>Chapter 2 on Structures; the introduction and the hospital core elements<br>Chapter 6 on Education and Training: The introduction and abbreviated tables for AMS competencies and workshop	**Introduction/Review of the toolkit and the interventions and education and training**<br>Chapter 3-5 on Interventions; plan, perform and assess AMS interventions<br>Chapter 6 on Education and Training: The introduction and abbreviated tables for AMS competencies and workshop.
**Acceptability:**<br>What can you tell me about the National Action Plan on AMR in (name of country)?<br>When was the NAP launched? Is it mutisectoral? Is there a steering committee?<br>Is stewardship a priority for the NAP?<br>How far has NAP advanced in terms of hospital-based stewardship?<br>What is missing entirely from the NAP?	**Acceptability:**<br>What can you tell me about the National Action Plan (NAP) on AMR in (name of country)?<br>Are you engaged with the NAP? How?<br>Is stewardship a priority for the NAP?<br>How far has NAP advanced in terms of hospital-based stewardship?<br>How well does the NAP cover your needs in relation to stewardship at (name of hospital)?	**Acceptability:**<br>Are you aware of the National Action Plan (NAP) on AMR in (name of country)? If yes, what can you tell me about the National Action Plan (NAP) on AMR in (name of country)?<br>How well does the NAP cover yourneeds in relation to stewardship at (name of hospital)?<br>What structures and committees (e.g., IPC and DTC) are in place at (name of hospital) to support stewardship? What is your role in working with these committees?

How applicable are the National Core elements 4 key thematic areas:<br>National plan and strategy;<br>Regulation and guidelines;<br>Awareness, training and competencies;<br>Supporting technologies and data;?<br>Which core elements would you change and why?<br>What core elements are missing to enable the implementation of national AMS programmes?<br>What core elements can be deleted as they are not applicable to your setting?<br>How do you think the stratification (basic/advanced) of the checklist items fits within the context of (name of country)?	What is missing entirely from the national-level NAP document?<br>What structures and committees are in place at (name of hospital) to support stewardship?<br>How applicable are the Hospital Core Elements 6 key thematic areas:<br>Senior Hospital Leadership;<br>Accountability and Responsibilities;<br>Available Expertise;<br>Education and Practical Training;<br>Monitoring and Surveillance;<br>Reporting and Feedback;?<br>Which core elements would you change and why?<br>What core elements are missing to enable the implementation of stewardship programming in this hospital? In other hospitals in (name of country)?<br>What core elements can be deleted as they are not applicable to your setting?<br>How do you think the stratification (basic/advanced) of the checklist items fits within the context of your hospital?	How applicable are the AMS interventions in chapter 3-5 and the thematic areas:<br>Planning AMS interventions;<br>Performing AMS interventions;<br>Assessing AMS intervention including Monitoring and Surveillance, andReporting and Feedback;<br>Would you change anything in relation to planning? Performing? Assessing? Why?<br>Is anything missing in this section of the toolkit that would enable the implementation of stewardship programming in your hospital? Should anything be deleted as they are not applicable to your setting?<br>How do you think the the improvement model would work in your setting?
**Demand:**<br>How would you prioritize the need for a comprehensive toolkit on AMS to support AMS policies and programs in (name ofcountry)?<br>How do you see the different national core elements being used?<br>Can you provide some examples of what you have in place or are planning for each of the 4 topics in the core elements (independent of whether they are already implemented or not)?<br>What do you think will facilitate or be barriers to the implementation of these core elements ? Which	**Demand:**<br>How would you priortize the need for a comprehensive toolkit on AMS to support AMS policies and programs in (name of country)?<br>How do you see the different hospital core elements being used?<br>Can you provide some examples of what you have in place or are planning for each of the 6 topics in the hospital core elements (independent of whether they are already implemented or not),<br>What do you think will facilitate or be barriers to the implementation of these core elements ? Which	**Demand:**<br>How would you priortize the need for a toolkit on AMS to support AMS policies, guidelines and programs in (name of hospital)?<br>How do you see the interventionsents being used?<br>Can you provide some examples of what you already can do in your hospital?<br>What do you think will facilitate or be barriers to the implementation of further stewardship interventions as mentioned in chapter 4?<br>Which barriers are linked to staffing, which to skills, which to

barriers are linked to staffing, which to skills, which to attitudes, which to data availablity and data systems?<br><br>What role do the core elements themselves play, and what is due to in-country factors?	barriers are linked to staffing, which to skills, which to attitudes, which to data availablity and data systems?<br><br>What role do the core elements themselves play, and what is due to in-country and hospital-specific factors?	attitudes, which to data availablity and data systems?
**Practicality:**<br><br>How suitable/practical do you find the core elements for implementation in limited resource settings?<br><br>How practical is the stepwise approach recommended for the implementation of the core elements in (name of country)?<br><br>What do you think are possible facilitators and barriers for using this stepwise approach in implementation of the toolkit?	**Practicality:**<br><br>How suitable/practical do you find the core elements for implementation in limited resource settings?<br><br>How practical is the stepwise approach recommended for the implementation of the core elements in your hospital?<br><br>What do you think are possible facilitators and barriers for using this stepwise apporach in implementation of the toolkit?	**Practicality:**<br><br>How suitable/practical do you find the interventions for implementation in limited resource settings?<br><br>How practical is the stepwise approach recommended for the implementation of the interventions in your hospital?<br><br>What do you think are possible facilitators and barriers for using this stepwise approach in implementation of the toolkit?
**Integration:**<br><br>How well do the toolkit`s national core elements fit into other national health plans, legislation, and the health system of (name of country)?<br><br>Can you provide some examples of where the core elements do not fit so well? Why do you think that is the case?<br><br>Can you also provide some examples of where the core elements fit well? Why do you think that is the case?<br><br>How could some/all the core elements be changed to be easier to integrate into the health system and existing AMS plans?	**Integration:**<br><br>How well do the toolkit`s hospital core elements fit into AMS within (name of hospital)?<br><br>How would you prioritize the implementation of the hospital core elements for (name of hospital) based on the current status of AMS here?<br><br>Can you provide some examples of where the core elements do not fit so well? Why do you think that is the case?<br><br>Can you also provide some examples of where the core elements fit well? Why do you think that is the case?<br><br>How could some/all the core elements be changed to be easier to integrate into the hospital and existing AMS plans (additions, deletions, edits)?	**Integration:**<br><br>How well do the toolkit`s chapters on interventions fit into AMS plan for (name of hospital)?<br><br>How would you prioritize the implementation of the interventions for (name of hospital) based on the current status of AMS here?<br><br>Can you provide some examples of where the interventionsdo not fit so well? Why do you think that is the case?<br><br>Can you also provide some examples of where the core elements fit well? Why do you think that is the case?<br><br>How could some/all the interventions be changed to be easier to integrate into the hospital and existing AMS plans?
**Adaptability:**	**Adaptability:**	**Adaptability:**

How do you see the core elements being adapted to (name of country) to better fit your context?<br><br>Can you provide some examples of how you could adapt the core elements to your country`s context?<br><br>Can you provide some examples of which core elements you could not adapt to your country`s context?<br><br>What do you think are possible facilitators and barriers adapting the core elements to your country context?	How do you see the core elements being adapted to (name of hospital) to better fit your context?<br><br>Can you provide some examples of how you could adapt the core elements to your hospital`s context?<br><br>Can you provide some examples of which core elements you could not adapt to your hospital`s context?<br><br>What do you think are possible facilitators and barriers adapting the core elements to your hospital's context?	How do you see the interventions better fitting your context?<br><br>Can you provide some examples of how you could adapt the interventions to your hospital`s context?<br><br>Can you provide some examples of which interventions you could not adapt to your hospital`s context?<br><br>What do you think are possible facilitators and barriers adapting the interventions to your hospital's context?
**Implementation and dissemination:**<br><br>What are the main issues that have to be addressed for the National core elements to be implemented in (name of country)?<br><br>What factors promote their implementation, both with regard to how the core elements are written and presented but also with regard to your country specific context?<br><br>What resources exist or are needed to promote implementation and sustainability of the national core elements?<br><br>What are possible facilitators and barriers for implementation and sustained use of the national core elements in (name of country)?	**Implementation and dissemination**:<br><br>What are the main issues that have to be addressed for the hospital core elements to be implemented in this hospital?<br><br>What factors promote their implementation, both with regard to how the core elements are written and presented but also with regard to your hospital's specific context?<br><br>What resources exist or are needed to promote implementation and sustainability of the hospital core elements?<br><br>What are possible facilitators and barriers for implementation and sustained use of the core elements in this hospital?	**Implementation and dissemination:**<br><br>What are the main issues that have to be addressed for the interventions to be implemented in this hospital?<br><br>What factors promote their implementation, both with regard to how the chapters on interventions are written and presented but also with regard to your hospital's specific context?<br><br>What resources exist or are needed to promote implementation and sustainability of the interventions ?<br><br>What are possible facilitators and barriers for implementation and sustained use of the interventions in this hospital?
**Education and training** (reintroduce the section of Chapter 6 provided to the respondent)<br><br>**Competency Framework:**<br><br>What factors would facilitate planning and implementation of a competency[1] framework to support education and training in (name of country)?	**Education and training** (reintroduce the section of Chapter 6 provided to the respondent)<br><br>**Competency Framework:**<br><br>What factors would facilitate planning and implementation of a competency framework to support education and training in this hospital?	**Education and training** (reintroduce the section of Chapter 6 provided to the respondent)<br><br>**Education and training delivery**: A wide variety of settings and opportunities are discussed in the toolkit in relation to providing education and training, e.g., "on-

[1] Compentencies are sets of behaviours that are instrumental in the delivery of better prescribing

What barriers might exist to planning and implementation of a competency framework?<br><br>**Education and training delivery:** A wide variety of settings and opportunities are discussed in the toolkit in relation to providing education and training, e.g., "on-the-job", "just-in-time", e-learning options.<br><br>What factors would facilitate implementation of these different options?<br><br>What barriers might exist in terms of implementation of these different options?<br><br>**Levels of competency:** (basic, competent/intermediate, advanced)<br><br>Are these levels adequate in terms of planning and implementing education and training programs in (name of country)? Are there additional levels that should be included?<br><br>How might these levels be adapted for implementation in (name of country)?<br><br>**Core topics:** (Introduction to AMR, Antibiotics, Microbiology, Clinical Syndromes, AMS, Stewardship Assessment)<br><br>Are there additional topics which should be included?<br><br>Do the tables provide the necessary detail regarding specific content relevant to each topic? What other information is needed to support implementation of training and eduction?<br><br>What factors might facilitate implementation of training and education on these topics? What barriers exist that would affect implementation?<br><br>**Resources:**	What barriers might exist to planning and implementation of a competency framework?<br><br>**Education and training delivery**: A wide variety of settings and opportunities are discussed in the toolkit in relation to providing education and training, e.g., "on-the-job", "just-in-time", e-learning options.<br><br>What factors would facilitate implementation of these different options?<br><br>What barriers might exist in terms of implementation of these different options?<br><br>**Levels of competency:** (basic, competent/intermediate, advanced)<br><br>Are these levels adequate in terms of planning and implementing education and training programs in this hospital? Are there additional levels that should be included?<br><br>How might these levels be adapted for implementation in this hospital?<br><br>**Core topics**: (Introduction to AMR, Antibiotics, Microbioogy, Clinical Syndromes, AMS, Stewardship Assessment)<br><br>Are there additional topics which should be included?<br><br>Do the tables provide the necessary detail regarding specific content relevant to each topic? What other information is needed to support implementation of training and eduction?<br><br>What factors might facilitate implementation of training and education on these topics? What barriers exist that would affect implementation?<br><br>**Resources:**<br><br>What institutions (e.g., professional schools, professional organizations) can provide support for implementation of training and	the-job", "just-in-time", e-learning options.<br><br>What factors would facilitate implementation of these different options?<br><br>What barriers might exist in terms of implementation of these different options?<br><br>**Levels of competency:** (basic, intermediate, advanced)<br><br>Are these levels adequate in terms of planning and implementing education and training programs in this hospital? Are there additional levels that should be included?<br><br>How might these levels be adapted for implementation in this hospital?<br><br>**Core topics**: (Introduction to AMR, Antibiotics, Microbioogy, Clinical Syndromes, AMS, Stewardship Assessment)<br><br>Are there additional topics which should be included?<br><br>Do the tables provide the necessary detail regarding specific content relevant to each topic? What other information is needed to support implementation of training and eduction?<br><br>What factors might facilitate implementation of training and education on these topics? What barriers exist that would affect implementation?<br><br>**Resources:**<br><br>What institutions (e.g., professional schools, professional organizations) can provide support for implementation of training and education programs? What human resources can support implementation of training and education programs?<br><br>What facilitators and barriers exist in relation to resources for

What institutions (e.g., professional schools, professional organizations) can provide support for implementation of training and education programs? What human resources can support implementation of training and education programs?<br><br>What facilitators and barriers exist in relation to resources for implementation of education and training?	education programs? What human resources can support implementation of training and education programs?<br><br>What facilitators and barriers exist in relation to resources for implementation of education and training?	implementation of education and training?
**Closing general questions**<br><br>What governance structure currently exists to implement the NAP and who would be responsible for implementing AMS programmes (the national core elements) and education and training components of the toolkit? What role might you play in implementation of the toolkit?<br><br>In terms of the toolkit document:<br><br>How do you find the proportion of text, tables and illustrations in the toolkit?<br><br>What is your opinion of the overall organization of the toolkit?<br><br>How complicated do you find the text? Are there specific areas that are more or less easy to understand? How should it be changed (additions, edits, deletions)?	**Closing general questions**<br><br>What structures and human resources currently exist to implement AMS within this hospital? Who/what institutional organizations/committees would be responsible for implementing the hospital core elements and education and training components of the toolkit?<br><br>What role might you play in implementation of the toolkit?<br><br>In terms of the toolkit document,<br><br>How do you find the proportion of text, tables and illustrations in the toolkit?<br><br>What is your opinion of the overall organization of the toolkit?<br><br>How complicated do you find the text? Are there specific areas that are more or less easy to understand? How should it be changed?	**Closing general questions**<br><br>What structures and human resources currently exist to implement AMS within this hospital?<br><br>What role might (physicians, nurese, pharmacists, microbiologists/laboratory staff - - [**choose based on respondent(s)**] play in implementation of the toolkit?<br><br>In terms of the toolkit document,<br><br>How do you find the proportion of text, tables and illustrations in the toolkit?<br><br>What is your opinion of the overall organization of the toolkit?<br><br>How complicated do you find the text? Are there specific areas that are more or less easy to understand? How shouldit be changed?
